# Functional Alteration and Differential Expression of the Bitter Taste Receptor T2R38 in Human Paranasal Sinus in Patients with Chronic Rhinosinusitis

**DOI:** 10.3390/ijms24054499

**Published:** 2023-02-24

**Authors:** Kota Takemoto, Luga Santo Lomude, Sachio Takeno, Tomohiro Kawasumi, Yukako Okamoto, Takao Hamamoto, Takashi Ishino, Yuki Ando, Chie Ishikawa, Tsutomu Ueda

**Affiliations:** Department of Otorhinolaryngology, Head and Neck Surgery, Graduate School of Biomedical Sciences, Hiroshima University, Hiroshima 734-8551, Japan

**Keywords:** bitter taste receptor (T2R), T2R14, T2R38, chronic rhinosinusitis (CRS), eosinophils, ciliated cells, nitric oxide (NO), single nucleotide polymorphism (SNP)

## Abstract

The bitter taste receptors (T2Rs) expressed in human sinonasal mucosae are known to elicit innate immune responses involving the release of nitric oxide (NO). We investigated the expression and distribution of two T2Rs, T2R14 and T2R38, in patients with chronic rhinosinusitis (CRS) and correlated the results with fractional exhaled NO (FeNO) levels and genotype of the T2R38 gene (TAS2R38). Using the Japanese Epidemiological Survey of Refractory Eosinophilic Chronic Rhinosinusitis (JESREC) phenotypic criteria, we identified CRS patients as either eosinophilic (ECRS, n = 36) or non-eosinophilic (non-ECRS, n = 56) patients and compared these groups with 51 non-CRS subjects. Mucosal specimens from the ethmoid sinus, nasal polyps, and inferior turbinate were collected from all subjects, together with blood samples, for RT-PCR analysis, immunostaining, and single nucleotide polymorphism (SNP) typing. We observed significant downregulation of T2R38 mRNA levels in the ethmoid mucosa of non-ECRS patients and in the nasal polyps of ECRS patients. No significant differences in T2R14 or T2R38 mRNA levels were found among the inferior turbinate mucosae of the three groups. Positive T2R38 immunoreactivity was localized mainly in epithelial ciliated cells, whereas secretary goblet cells generally showed lack of staining. The patients in the non-ECRS group showed significantly lower oral and nasal FeNO levels compared with the control group. There was a trend towards higher CRS prevalence in the PAV/AVI and AVI/AVI genotype groups as compared to the PAV/PAV group. Our findings reveal complex but important roles of T2R38 function in ciliated cells associated with specific CRS phenotypes, suggesting the T2R38 pathway as a potential therapeutic target for promotion of endogenous defense mechanisms.

## 1. Introduction

Bitter taste receptors (T2Rs) are chemosensory proteins belonging to the G protein-coupled receptor (GPCR) superfamily [1]. Humans are known to express 25 different T2Rs, which are variously activated by more than one thousand compounds [2]. The bitter molecules include plant extracts (alkaloids, terpenoids, flavonoids, etc.), bacterial products, and synthetic chemical compounds [3,4]. Numerous studies conducted in recent years have shown expression of bitter receptors in a wide variety of extraoral tissues. These receptors are considered to perform diverse and important physiological functions apart from their better-understood role in taste [5,6,7]. The bitter taste receptors in the human airways are known to elicit innate immune responses and eradicate airborne pathogens [8,9,10]. Recently, T2Rs have been studied for their roles in sinonasal immunity and their involvement in chronic rhinosinusitis (CRS) pathophysiology [11,12,13,14]. Epithelial ciliated cells express at least one bitter receptor (T2R38, encoded by the TAS2R38 gene) that recognizes foreign bacteria, viruses, or fungi. The downstream pathways trigger immune defenses leading to the release of nitric oxide (NO), which plays crucial roles in the maintenance of physiological homeostasis with bactericidal effects [15]. T2R14 is one of the highly expressed T2Rs in the upper respiratory epithelial cells of humans [16] and is activated by plant flavones, *Pseudomonas aeruginosa* quinolones [17], and many pharmaceutical drugs [18,19].

Chronic rhinosinusitis (CRS) is a common disease with a considerable social burden. It is defined as the symptomatic inflammation of the sinonasal mucosa with evidence of inflammation on exam and/or imaging lasting more than 12 weeks [20]. CRS patients are categorized into two subtypes based on their phenotypic features: those with nasal polyps (CRSwNP) and those without (“sans”, CRSsNP) [21]. Eosinophilic chronic rhinosinusitis (ECRS) is a subgroup of CRSwNP that is associated with severe eosinophilic infiltration and intractable social burden according to the Japanese Epidemiological Survey of Refractory Eosinophilic Chronic Rhinosinusitis (JESREC) [22]. ECRS is a predominantly type 2-mediated inflammatory disease characterized by the presence of increased levels of cytokines including interleukins (IL) -4, -5, -13, -25, and -33, as well as thymic stromal lymphopoietin (TSLP) [23]. Persistent symptoms include nasal blockage, nasal discharge, and olfactory dysfunction, with patients frequently reporting an altered sense of taste [24,25,26]. As a CRS symptom, a loss of taste has been drawing increasing attention and is believed to be associated with a loss of smell [27,28]. However, studies have yet to elucidate the cellular mechanisms related to taste receptor function and its downstream immunologic effects, and to associate these features with the different CRS subsets.

In the present study, we hypothesized the possible roles of T2Rs in CRS in relation to underlying pathophysiologic mechanisms that translate into clinical phenotypes, i.e., non-ECRS and ECRS. For this purpose, we investigated the gene expression level and protein localization of T2R14 and T2R38 in the inferior turbinate and paranasal sinus mucosae of CRS patients and controls and assessed their relationship with the levels of fractional exhaled NO (FeNO). In addition, we examined whether differences in TAS2R38 alleles at the pertinent chromosomal location influenced CRS prevalence in the study population. The T2R38 isoform is the most well-known and well-characterized T2R, the haplotypes of which have common polymorphisms, one encoding a functional receptor and the other encoding a nonfunctional one [29,30,31]. The functional allele of “high tasters” contains the proline-alanine-valine (PAV) sequence, whereas “low tasters” have the alanine-valine-isoleucine sequence (AVI). Our results herein suggest the potential value of T2R38 as a therapeutic target for the promotion of endogenous immune responses in CRS patients involving increased NO production.

## 2. Results

### 2.1. Background and Characteristics of Subjects in the Study

The background and clinical characteristics of the study population are summarized in Table 1. We divided the 92 CRS patients into two groups based on the JESREC criteria for diagnosing ECRS [22]: namely, a non-ECRS group (n = 56) and an ECRS group (n = 36). No significant difference was found among the three groups in the baseline data of gender, allergic rhinitis (AR) co-morbidity, or body mass index (BMI). Mean age was significantly higher in the non-ECRS group (*p* < 0.001) and the ECRS group (*p* < 0.01) than in the control group. Significant differences were observed between the non-ECRS and ECRS groups in the proportion of asthma co-morbidity, the degree of blood and tissue eosinophils, and the severity of CT scores. Further, the non-ECRS patients showed a significantly lower level of blood eosinophils than the control subjects.

**Figure 1:**
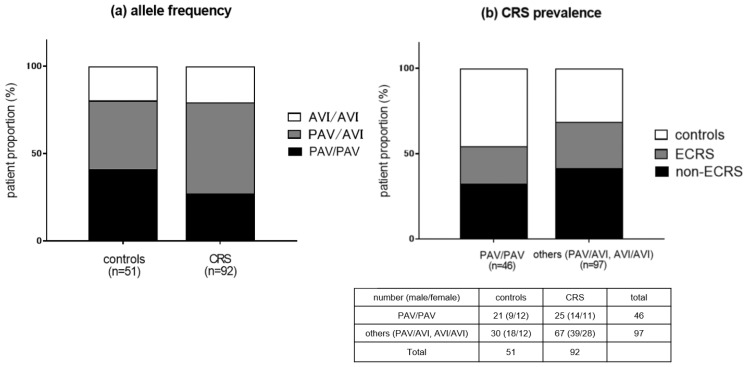
(**a**) Proportions of the PAV/PAV allele and other alleles (PAV/AVI and AVI/AVI) in the control and CRS groups. (**b**) CRS prevalence classified by TAS2R38 gene polymorphisms (PAV/PAV vs. PAV/AVI and AVI/AVI alleles).

We examined single nucleotide polymorphisms (SNPs) of the TAS2R38 gene in the study population. The proportion of the PAV/PAV allele and other alleles (PAV/AVI and AVI/AVI) in the controls and CRS (ECRS + non-ECRS) groups are shown in Figure 1a. The PAV/PAV proportion tended to be higher in the control group than in the CRS group (41.1% vs. 27.1%), although the difference was not statistically significant (*p* = 0.085). The CRS prevalence classified by TAS2R38 gene polymorphisms is shown in Figure 1b. There was a trend towards higher CRS prevalence in the non-PAV/PAV genotype group as compared to the PAV/PAV group (69% vs. 54.3%). However, the difference was not significant (*p* = 0.085). As for gender difference, the PAV/PAV proportion in females tended to be higher than in males in the control group, although the difference was not statistically significant (*p* = 0.265). No significant differences in gender proportion for each SNP allele was found in the CRS prevalence.

### 2.2. Target Genes Expression in Sinonasal Mucosa and Nasal Polyps

**Figure 2:**
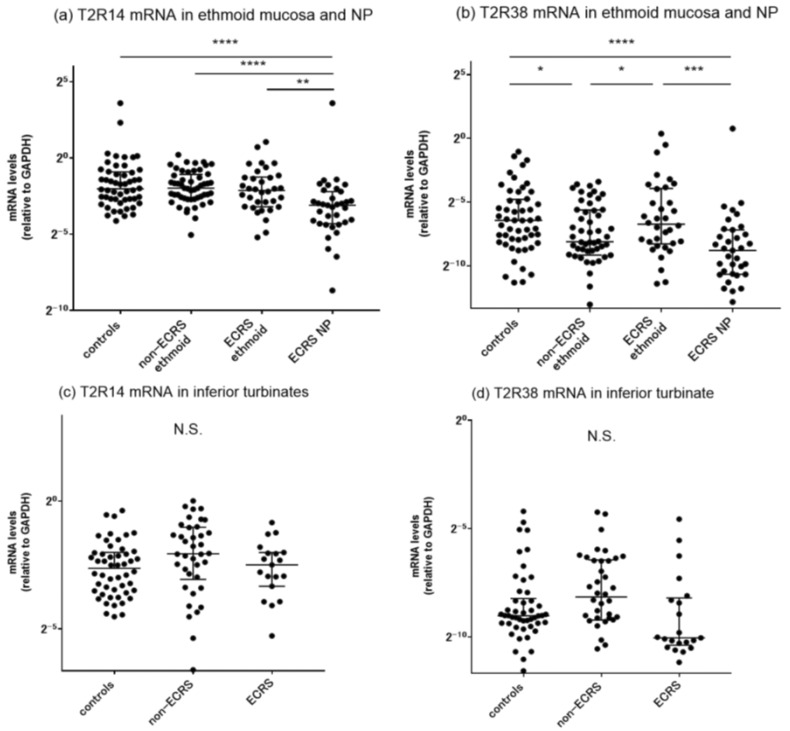
Comparison of mRNA expression in ethmoid sinus, nasal polyps, and inferior turbinate mucosae from controls, non-ECRS, and ECRS patients as detected by RT-PCR. (**a**,**c**) T2R14 and (**b**,**d**) T2R38 mRNA levels were quantitatively normalized to the GAPDH mRNA levels. * *p* < 0.05; ** *p* < 0.01; *** *p* < 0.001; **** *p* < 0.0001. Center lines, median values; error bars: interquartile ranges; N.S., not significant.

**Figure 3:**
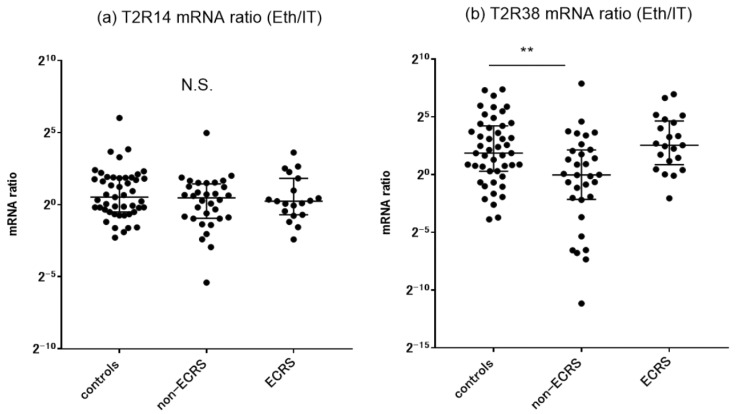
Comparison of the mRNA expression ratios of ethmoid sinus to inferior turbinate mucosa (Eth/IT ratio) for each subject in the control, non-ECRS, and ECRS groups. (**a**) T2R14 and (**b**) T2R38. ** *p* < 0.01. Center lines, median values; error bars, interquartile ranges; N.S., not significant.

Previous studies have reported that human bitter taste receptors are expressed in human upper airway tissues and that the density of these receptor proteins is higher in the ethmoid sinus than in the nasal cavity [16]. We therefore sought to evaluate T2R mRNA expression in different areas of the sinonasal mucosae in both CRS and control groups. The mRNA levels of T2R14 and T2R38 in the ethmoid sinus mucosa, nasal polyps, and inferior turbinate mucosa were assessed by quantitative RT-PCR (Figure 2). Nasal polyp specimens in ECRS patients showed a significant downregulation of T2R14 mRNA expression compared to the ethmoid mucosa samples across all groups. We also observed significant downregulation of T2R38 mRNA levels in the ethmoid mucosa of non-ECRS patients and in the nasal polyps of ECRS patients. There was no significant difference in T2R14 and T2R38 mRNA levels among the inferior turbinate mucosae of the three groups. We also plotted the ratio of mRNA expression levels of ethmoid sinus mucosa to inferior turbinate mucosa (Eth/IT ratio) for each subject (Figure 3). There was no significant difference in the Eth/IT ratios for T2R14 among the groups. In contrast, the non-ECRS patients showed a significantly lower Eth/IT ratio for T2R38, reflecting the lower mRNA levels in the ethmoid sinus area.

### 2.3. Immunohistochemical Distribution of T2R14 and T2R38 Proteins

**Figure 4:**
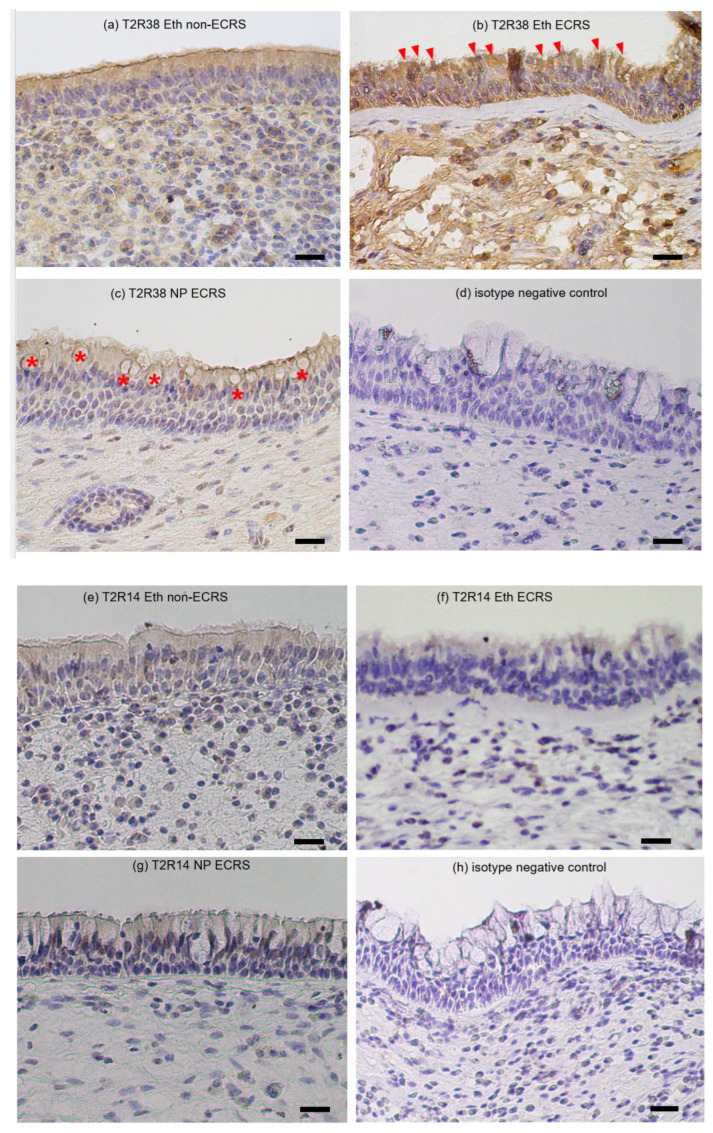

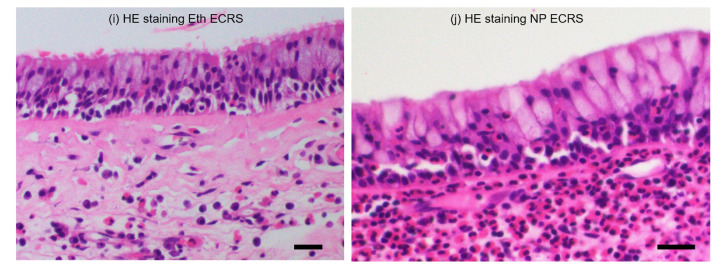
Representative immunohistological images showing T2R38 (**a**–**c**) and T2R14 (**e**–**g**) protein distribution in ethmoid sinus and nasal polyp mucosa sampled from non-ECRS and ECRS patients. Isotype negative controls for T2R38 (**d**) and T2R14 (**h**). HE staining images of (**i**) ethmoid mucosa and (**j**) nasal polyps obtained from the same ECRS patient. Epithelial ciliated cells stained positive for T2R38. Higher rates of staining were observed in epithelial cells in ECRS patients along the ciliated luminal surface (Figure 4b, arrowheads). In contrast, secretary goblet cells in the epithelial layer generally show a lack of staining (Figure 4c, asterisks). Positive T2R14 immunoreactivity is localized mainly to epithelial cells with cytoplasmic staining. Scale bar: 20 μm.

Since transcriptional changes in T2R38 were associated with CRS pathology and clinical manifestations, we examined the sinus tissue distribution of T2R14 and T2R38 proteins in representative cases. Figure 4 provides immunohistological images of the distributions of T2R38- and T2R14-positive cells in the ethmoid sinus and nasal polyp mucosae. In the non-ECRS group, intense inflammatory cell infiltration with neutrophils and lymphocytes dominated the ethmoid mucosa on conventional histological examination. By contrast, dense eosinophil infiltration was observed in the ECRS group (Figure 4i,j). Positive T2R38 immunoreactivity was localized mainly in epithelial ciliated cells along the luminal surface. The ethmoid specimens from the patients in the ECRS group generally showed higher rates of T2R38-positive cells throughout the epithelial area as compared to those from the non-ECRS group, with ciliated cells being predominant (Figure 4a,b). In contrast, secretary goblet cells that were widely scattered in the epithelial layer of nasal polyp specimens showed a lack of staining (Figure 4c). The degree of T2R14 staining appeared to be identical among the specimens of the three groups.

### 2.4. FeNO Levels in CRS Patients

**Figure 5:**
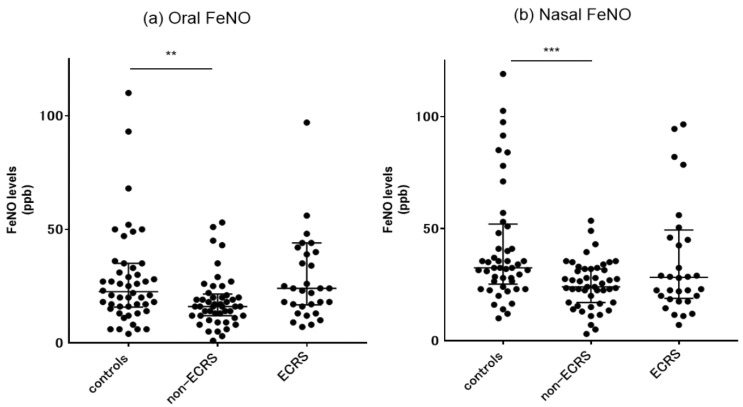
(**a**) Oral FeNO and (**b**) nasal FeNO levels in control subjects and non-ECRS and ECRS patients. ** *p* < 0.01; *** *p* < 0.001; FeNO, fractional exhaled nitric oxide. Center lines, median values; error bars, interquartile ranges.

Epithelial ciliated cells covering a large area of the human paranasal sinuses produce large amounts of NO, which plays a major role in airway physical defense by inducing increased ciliary beating [15]. T2R activation in response to T2R38-specific agonists results in the release of NO by means of calcium-dependent activation of constitutive nitric oxide synthase (NOS), contributing substantially to antibacterial defense mechanisms [32]. We therefore performed measurements of oral and nasal FeNO values in each group as a possible surrogate marker of T2R functional activities. As shown in Figure 5, the median oral FeNO levels were 22.5 (interquartile range; 15.7–35) ppb in control subjects, 16.0 (12–21.5) ppb in non-ECRS patients, and 24 (16.8–44) ppb in ECRS patients. The median nasal FeNO levels were 32.5 (25.3–52) ppb in control subjects, 24 (17–32) ppb in non-ECRS patients, and 28.3 (18.9–49.4) ppb in ECRS patients. Compared with the control group, the non-ECRS patients showed significantly lower both oral and nasal FeNO levels.

## 3. Discussion

The nasal cavity and paranasal sinuses are frontline defense systems of the human respiratory tract, where immunologic responses occur against various aerosolized pathogens contaminating inhaled air [10,33]. CRS is considered a category of heterogenous syndromes resulting from dysfunctional interactions between various environmental factors and the host immune system [20]. The complexity of CRS pathology has been evidenced by numerous research efforts aimed at recognizing more detailed endotypes, i.e., those defined by the presence of particular patterns of immune cells or biomarkers, and at developing optimal treatment modalities for each subset of patients [23,34,35,36,37].

As a CRS symptom, loss or changes of taste have drawn attention and are reported to be associated with a loss of smell [25,26,27,28]. Although extraoral T2R receptor expression in sinonasal epithelium would not affect the ability of taste and flavor perception in CRS patients, the association between improvements in taste and smell is intriguing given that the latter has been shown to correlate with CRS disease severity [20,24,38]. Studies have yet to elucidate the cellular mechanisms related to taste receptor function and immunologic responses underlying the different CRS subsets. In this sense, bitter taste receptors have gained attention for their roles in sinonasal immunity and contributions to CRS pathophysiology [11,12,13,14]. So far, two different cell types in the sinonasal mucosa have been reported to express T2Rs: namely, epithelial ciliated cells and solitary chemosensory cells (SCCs) [39]. In the present study, we tried to assess the possible relationship between the expressions and distributions of representative T2Rs and CRS pathology, with emphasis on the CRS phenotypic classification, as a measure for innate immunity function. To date, there has been no study concerning the interplay of T2R38 functional expression and development of CRS with different phenotypes in the Japanese population. We also examined whether TAS2R38 gene polymorphisms caused subjects to be more susceptible to sinonasal infection through attenuated T2R38 function, because the receptor’s function is dictated by specific genetic polymorphisms [13,40]. Further, no previous report has directly examined the relationship between T2R expression levels and nasal FeNO concentrations, as well as the frequency of genetic variants of T2R38 in the Japanese population.

In this study, we found significantly decreased T2R38 mRNA levels in the ethmoid mucosae of non-ECRS patients and in the nasal polyps of ECRS patients. Nasal polyp specimens in ECRS patients also showed a significant downregulation of T2R14 mRNA expression. There was no significant difference in the T2R14 and T2R38 mRNA levels of the inferior turbinate mucosae among the groups. The results indicate differences in anatomical structure and physiological function between the inferior turbinate and the ethmoid sinus mucosa, with the latter being intimately involved in the development of CRS pathology. Chen et al. reported that expression of T2Rs was higher in the ethmoid sinus than in other locations of the nasal cavity (inferior turbinate, middle turbinate, and nasal septum) [16]. In contrast with rhinitis, whose lesions are restricted to the nasal cavity with the inferior turbinate of particular importance [41], the paranasal sinuses and especially the ethmoid sinus are the major areas affected in CRS. Biopsies obtained from the ethmoid sinus may serve as the best location for the functional study of upper airway taste receptors in humans.

Interestingly, nasal polyps showed significantly lower mRNA levels of T2R14 and T2R38 than ethmoid sinus mucosae obtained from the same patients. Our immunohistochemical studies indicate that positive T2R immunoreactivity was localized mainly in epithelial ciliated cells along the luminal surface, whereas secretary goblet cells predominantly observed as epithelial non-ciliated components of nasal polyp specimens showed a lack of staining [20,42]. So far, there are no data available to directly compare T2R gene expression between the ethmoid sinus and nasal polyps in human CRS. Our results are supported by previous reports that T2R proteins are found exclusively within ciliated cells [43]. Recent immunohistochemical analysis reported a significant difference in T2R38 protein levels between both CRSsNP and CRSwNP patients and healthy controls [44]. We consider that the decreased expression of T2R38 in nasal polyps is attributable to the fact that nasal polyps contain higher proportions of secretory cells with fewer ciliated cells.

T2R activation results in the release of cellular NO and the increase in ciliary beat frequency (CBF), both of which contribute to the maintenance of airway physiological homeostasis [10,31]. T2R38 appears to be an essential mediator of sinonasal epithelial defense against respiratory bacterial infections [9,13,45]. When ciliated cells were stimulated with known T2R38-specific agonists, such as the bitter-tasting synthetic compound phenylthiocarbamide (PTC), they exhibited calcium-dependent activation of nitric oxide synthase (NOS) [13]. The robust NO production leads to cellular protein phosphorylation by protein kinase G (PKG) and increases ciliary beat frequency to facilitate the mucous movement out of the airway [19]. T2R38 also detects bacterial products such as acyl-homoserine lactones (AHLs) produced by *P. aeruginosa* [8,9,45] and activates NO production specifically via the NOS3 isoform localized on the cilia [42]. The NO that is generated diffuses into the airway surface liquid (ASL) and plays roles in anti-bacterial defense mechanisms [17,46].

We found that both oral and nasal FeNO levels in non-ECRS patients were significantly lower than those of control subjects and were associated with decreased T2R38 mRNA expression levels in the ethmoid mucosae of such patients. The non-ECRS patients also showed a significant decrease in the Eth/IT ratio for T2R38, which might primarily reflect lower mRNA levels in the ethmoid sinus area. Further, immunostaining revealed that T2R38 expression in the ethmoid sinus mucosa in the non-ECRS patients generally showed lower degrees of T2R38-positive cells throughout the epithelial area compared to ECRS patients. The results support our hypothesis that the onset and persistence of CRS morbidity might be partly triggered by decreased NO production due to attenuated expression of T2R38 in the middle meatus area, leading to the vulnerability of defense systems against airborne pathogens. The role of nasal FeNO in CRS patients has been a matter of debate due to its multiple origin, with contributions from both the paranasal sinus cavities and the inflamed sinonasal mucosa [15,47]. Non-ECRS patients generally showed lower FeNO levels as a result of occluded paranasal sinus ventilation and damaged ciliated epithelia [48]. On the other hand, several attempts have been made to measure nasal NO levels as a marker for assessing the severity of type 2 inflammation with tissue eosinophils of ECRS patients [48,49]. The present results of FeNO levels are compatible with our previous studies from different patient populations [48]. The treatment of CRS may restore both the NOS expression of the sinus ciliated cells and the ability of NO to pass through the paranasal sinus ostia. This is particularly important in cases of non-ECRS patients with a limited area of sinus disease [50]. Anyway, further research is required to elucidate how the post-surgery recovery process of sinus ciliary epithelial cells is functionally related to recovery of T2R38 expression levels that leads to NO production with morphological integrity.

The present study also suggests that development of T2R38 stimulatory pharmaceutical components combined with delivery devices might provide an attractive therapeutic option to augment natural host responses in the treatment of CRS. The use of secreted bacteria-derived products for treatment in adult CRS patients is empirically recommended and is also supported by the EPOS 2020 with level 1b evidence [20,51]. On the other hand, no significant difference was observed in T2R14 expression levels among the groups. T2R14 is one of the highly expressed T2R isoforms in nasal and lung epithelial cells [52]. T2R14 also responds to AHL quorum-sensing molecules produced by Gram-negative bacteria [17]. Further study is required to understand how different T2R isoforms’ responses are modulated within the inflammatory milieu of CRS pathology.

The most well-known and well-characterized example is the T2R38 isoform [7,29]. The TAS2R38 gene has two common polymorphisms, one encoding a functional receptor and the other encoding a nonfunctional receptor [45]. The differences in the resulting proteins are at amino acid positions 49, 262, and 296. The functional T2R38 receptor contains proline (P), alanine (A), and valine (V) residues, while nonfunctional T2R38 contains alanine (A), valine (V), and isoleucine (I). Loss of the valine in the AVI variant is responsible for the impairment of receptor activation [30,53]. The resulting haplotypes influence the perception of bitter taste: PAV–PAV as “supertasters,” PAV–AVI as variable or intermediate-level tasters, and AVI–AVI as “nontasters”. In the study of TAS2R38 genotypes, we found a tendency for a higher prevalence of CRS in subjects with PAV/AVI and AVI/AVI SNP alleles as compared to subjects with the PAV/PAV allele. The proportion of PAV/PAV T2R38 genotype also tended to be lower in CRS patients than in controls. Genetic polymorphisms are common within the taste receptors [9]. T2Rs are genetically diverse, which helps to explain the wide variety of taste preferences both within and among cultures [30]. In addition, several recent linkage studies have demonstrated associations of T2R isoforms genetics with CRS morbidity, including TAS2R13, TAS2R19, TAS2R38, and TAS2R49 [11,12]. We examined associations between the TAS2R38 polymorphisms and phenotypic CRS prevalences in the Japanese population due to the broad clinical implications of extraoral expression of T2R38. These polymorphisms are reported to be distributed in a nearly Mendelian ratio in Caucasian populations [54]. Adappa et al. reported that distributions of AVI/AVI, AVI/PAV, and PAV/PAV alleles were 37%, 54%, and 8.5%, respectively, in CRS patients in Caucasian populations compared with 29%, 51%, and 20%, respectively, in the general regional population in America [12]. Their results were comparable with ours: namely, in our study, AVI/AVI, AVI/PAV, and PAV/PAV alleles were 20.6%, 52.1%, and 27.1%, respectively, in CRS patients as compared to 19.6%, 39.2%, and 41.1% in the control subjects [9,52]. A series of these studies have highlighted the potential relevance of T2R38 in CRS. Individuals who express the fully functional PAV/PAV genotype are less likely to contract upper airway infection by gram-negative bacteria [11,40] or to require surgical intervention [12,55].

This study has several limitations. First, the study included a relatively small number of Japanese patients; hence, caution should be taken when extrapolating our results to other ethnic groups. Further prospective studies on a larger scale are requisite to elucidate the mucociliary clearance function via T2Rs activation. Second, we failed to examine the second sinonasal cell type that expresses bitter receptors, i.e., the solitary chemosensory cell (SCC), because these cells also modulate the epithelial innate immune system [32,39,56]. Third, the degree of functional expression of T2R38 and nasal FeNO levels is a partial element of recalcitrant CRS, which should be viewed in the context of other genetic and environmental influences yet to be clarified. For example, our previous study demonstrated that ECRS patients with genetically longer (CCTTT)n repeat polymorphisms had higher expressions of NOS2 mRNA in ethmoid sinus mucosae with higher FeNO levels in certain clinical manifestations [57].

In conclusion, we have demonstrated that changes in T2R38 expression levels and localization in the sinonasal pathway are associated with specific CRS phenotype (non-ECRS and ECRS) subsets, suggesting the T2R38 pathway as a potential therapeutic target to promote endogenous immune responses in CRS patients. This discovery is likely to have significant clinical impact immediately and to prompt further studies to define the T2R38 signaling pathways as well as to identify other T2Rs that similarly activate immune responses.

## 4. Materials and Methods

### 4.1. Study Design

We conducted a case–control study of 56 patients with non-ECRS and 36 patients with ECRS, all of whom underwent endoscopic sinus surgery. The diagnosis of sinus disease was based on the patient’s history, clinical symptoms, endoscopic findings, and computed tomography (CT) imaging. Patients with a previous sinus surgery were excluded. None of the patients had received topical or systemic steroids for ≥4 weeks prior to the surgery. The CT images were subjected to radiological grading using the Lund-Mackay system [58]. The diagnosis of AR was based on clinical history, presence of nasal symptoms together with positive nasal eosinophils, and positive allergen-specific IgE antibodies. JESREC scoring was used to differentiate ECRS from non-ECRS. The scores include 4 items: bilateral sinus disease, the presence of nasal polyps, the degree of eosinophilia in peripheral blood, and mucosal eosinophil count ≥70/high-power field (HPF) [22]. Fifty-one patients without sinus infection who underwent endonasal surgery served as controls. All controls had paranasal sinus mucosa of normal appearance and normal radiological findings.

Oral and nasal FeNO levels were measured before surgery using a handheld electrochemical analyzer (NObreath^®^, Bedfont Scientific Ltd., Rochester, UK) according to ATS/ERS guidelines [47]. For oral FeNO measurements, subjects were advised to exhale at a flow rate of 50 mL/s through a mouthpiece. For nasal FeNO measurements, subjects were instructed to exhale transnasally with their mouth closed into a nose adaptor as described elsewhere [59]. Each measurement was performed in triplicate, and the mean value was used for analysis.

### 4.2. RT-PCR Analysis

Mucosal specimens were obtained from the ethmoid sinus, nasal polyps (if any), and the inferior turbinate at the time of surgery. When CRS was present bilaterally, specimens were taken from both sides. The specimens were divided and either immersed in RNAlater^®^ solution (Ambion, Austin, TX, USA) for RT-PCR or fixed in 10% neutral buffered formaldehyde for immunohistochemistry. Quantitative PCR analysis was performed on an ABI Prisms 7300 system (Applied Biosystems, Foster City, CA, USA). Cellular RNA was isolated using RNeasy mini kits (Qiagen, Valencia, CA, USA). Total RNA was then reverse-transcribed to cDNA using a high-capacity RNA-to-cDNA kit (Applied Biosystems) according to the manufacturer’s instructions. Gene expression was measured on a real-time PCR system using TaqMan Gene Expression Assays (Thermo Fisher Scientific, Waltham, MA, USA). PCR primers specific for TAS2R14 (Hs00256800_s1) and TAS2R38 (Hs00604294_s1) were used (Thermo Fisher Scientific). Primers for GAPDH (Hs03929097_g1) were used as a reference. Amplifications of the PCR products were quantified by the number of cycles, and the results were analyzed using the comparative cycle threshold (Ct) method (2^−ΔΔCt^). The quantities of target gene expression are presented as relative rates compared to the expression of the reference gene (ratio: target gene/GAPDH expression).

### 4.3. Immunohistochemistry

The primary antibodies used were anti-human TAS2R14 rabbit polyclonal antibody (#PA020246; Cusabio, Houston, TX, USA) and anti-human TAS2R38 rabbit polyclonal antibody (#PA023155LA01HU; Cusabio). Surgical tissue specimens embedded in paraffin were sliced into 5 µm thick sections for immunostaining. For antigen retrieval, sections were immersed in Histo VT One (Nacalai Tesque, Kyoto, Japan) at 70 °C for 40 min. The slides were then incubated overnight at 4 °C with the primary antibodies. Color development was achieved using the streptavidin-biotin amplification technique (ChemMate EnVision kit; Dako, Glostrup, Denmark). Peroxidase activity was visualized by diaminobenzidine solution. Sections were counterstained with hematoxylin. Control specimens with IgG1 isotype control were used to verify that the nonspecific binding was not detectable. Consecutive sections were routinely stained with hematoxylin-eosin (HE) for the assessment of mucosal pathology and the degree of eosinophil infiltration.

### 4.4. Genotyping the SNP Polymorphism in the TAS2R38 Gene

Peripheral whole blood was collected from all patients for genotyping of the single nucleotide variation at rs10246939 in the TAS2R38 gene in chromosome 7. Genomic deoxyribonucleic acid (DNA) was extracted from blood using the PAXgene^®^ Blood DNA kit (Qiagen, Hilden, Germany). The genotypes were assessed using the TaqMan SNP genotyping assay (C___9506826_10, Thermo Fisher Scientific). Then, haplotypes (C/T) and diplotypes (C/C and T/T) were identified and recorded using Applied Biosystem^TM^ StepOnePlus^®^ Real-Time PCR systems (Applied Biosystems). We categorized subjects with two copies of the PAV allele (PAV/PAV) as high tasters and those with only one copy of the PAV allele (PAV/AVI) or no PAV alleles (AVI/AVI) as low tasters [11].

### 4.5. Data Analysis

Power and sample size calculations for the study design were performed based on data from previous studies of sinonasal T2R and NOS expression. The G*power program, version 3.1.9.6, was used for estimation (https://www.psychologie.hhu.de/arbeitsgruppen/allgemeine-psychologie-und-arbeitspsychologie/gpower.html (accessed on 2 January 2023)). For multiple comparisons, a screening of the data for differences was first carried out using the Kruskal-Wallis test. If the analysis gave a significant result, a further comparison was done by the Mann-Whitney U-test for the between-group analysis. Fisher’s exact test was used to compare qualitative data. *p*-values < 0.05 were considered significant.

All procedures contributing to this work complied with the ethical standards expressed in the Helsinki Declaration. The study protocol was approved by the Institutional Review Board at the Hiroshima University School of Medicine (Approval No. Hi-136-2). Written informed consent was obtained from all patients prior to their participation.

## Figures and Tables

**Table 1:** Background and baseline characteristics of the study population.

	Controls	Non-ECRS	ECRS
Number (male/female)	51 (27/24)	56 (35/21)	36 (18/18)
Age (mean ± SD)	44.7 ± 17	56.1 ± 15.7 †††	54 ± 11.3 ††
Allergic rhinitis (%)	35 (68.6%)	31 (55.4%)	28 (77.8%)
BMI (kg/mm^2^) (mean ± SD)	23.8 ± 3.7	23.2 ± 3.3	22.6 ± 3.5
Bronchial asthma (%)	6 (11.8%)	6 (10.7%)	19 (52.8%) ***
Blood eosinophils (%) (median, range)	3.6 (1.6–5.7)	1.9 (1.5–3.5) †	6.95 (5.5–9.6) ***
Tissue eosinophils (cells/HPF) (median, range)	4.6 (0.7–14)	5 (2.4–18.2)	117 (75–233) ***
CT score (mean ± SD)		7.27 ± 5.8	15 ± 5.2 ***

Data are shown as mean ± standard deviation (SD), median (range), or number (%). *** *p* < 0.001 vs. the other groups. † *p* < 0.05, †† *p* < 0.01, ††† *p* < 0.001 vs. the control group. ECRS, eosinophilic chronic rhinosinusitis; BMI, body mass index; HPF, high power field (x400); CT, computed tomography.

## Data Availability

The data presented in this study are available on reasonable request from the corresponding author.
